# Broca’s Area as a Pre-articulatory Phonetic Encoder: Gating the Motor Program

**DOI:** 10.3389/fnhum.2018.00064

**Published:** 2018-02-22

**Authors:** Valentina Ferpozzi, Luca Fornia, Marcella Montagna, Chiara Siodambro, Antonella Castellano, Paola Borroni, Marco Riva, Marco Rossi, Federico Pessina, Lorenzo Bello, Gabriella Cerri

**Affiliations:** ^1^Laboratory of Motor Control, Department of Medical Biotechnology and Translational Medicine, University of Milan, Humanitas Research Hospital, IRCCS, Milan, Italy; ^2^Neurosurgical Oncology Unit, Department of Oncology and Hemato-Oncology, University of Milan, Humanitas Research Hospital, IRCCS, Milan, Italy; ^3^Neuroradiology Unit and CERMAC, Vita-Salute San Raffaele University and IRCCS San Raffaele Scientific Institute, Milan, Italy; ^4^Department of Health Sciences, University of Milan, Milan, Italy; ^5^Neurosurgical Oncology Unit, Department of Medical Biotechnology and Translational Medicine, University of Milan, Humanitas Research Hospital, IRCCS, Milan, Italy; ^6^Cancer Neurosurgery Unit, Humanitas Research Hospital, IRCCS, Milan, Italy

**Keywords:** Broca’s area, speech, motor control, brain mapping, phonetic encoding

## Abstract

The exact nature of the role of Broca’s area in control of speech and whether it is exerted at the cognitive or at the motor level is still debated. Intraoperative evidence of a lack of motor responses to direct electrical stimulation (DES) of Broca’s area and the observation that its stimulation induces a “speech arrest” without an apparent effect on the ongoing activity of phono-articulatory muscles, raises the argument. Essentially, attribution of direct involvement of Broca’s area in motor control of speech, requires evidence of a functional connection of this area with the phono-articulatory muscles’ motoneurons. With a quantitative approach we investigated, in 20 patients undergoing surgery for brain tumors, whether DES delivered on Broca’s area affects the recruitment of the phono-articulatory muscles’ motor units. The electromyography (EMG) of the muscles active during two speech tasks (object picture naming and counting) was recorded during and in absence of DES on Broca’s area. Offline, the EMG of each muscle was analyzed in frequency (power spectrum, PS) and time domain (root mean square, RMS) and the two conditions compared. Results show that DES on Broca’s area induces an intensity-dependent “speech arrest.” The intensity of DES needed to induce “speech arrest” when applied on Broca’s area was higher when compared to the intensity effective on the neighboring pre-motor/motor cortices. Notably, PS and RMS measured on the EMG recorded during “speech arrest” were superimposable to those recorded at baseline. Partial interruptions of speech were not observed. Speech arrest was an “all-or-none” effect: muscle activation started only by removing DES, as if DES prevented speech onset. The same effect was observed when stimulating directly the subcortical fibers running below Broca’s area. Intraoperative data point to Broca’s area as a *functional gate* authorizing the phonetic translation to be executed by the motor areas. Given the absence of a direct effect on motor units recruitment, a direct control of Broca’s area on the phono-articulatory apparatus seems unlikely. Moreover, the strict correlation between DES-intensity and speech prevention, might attribute this effect to the inactivation of the subcortical fibers rather than to Broca’s cortical neurons.

## Introduction

Speech represents the unique human ability to translate thoughts and feelings in articulate sounds. This neural function has been historically attributed to a complex network involving, as essential components, the *pars opercularis* and *triangularis* (Brodmann Area BA44 and BA45, respectively) of the posterior Inferior Frontal Gyrus (IFG). Broca (1861) reported the lesion of this area as the distinguishing feature in brains of patients affected by permanent “speech loss,” therefore BA44-45, now called Broca’s area, was consecrated as an essential hub in the neural control of speech production (Berker et al., 1986; Amunts et al., 1999). The “speech loss” in Broca’s patients, described as the inability to *articulate language* (Berker et al., 1986), was termed *production* (or *Broca’s*) *aphasia*. The term “production” aphasia and its direct identification with a deficit in the executive articulation of speech sounds, lead to the logical but unsubstantiated conclusion that Broca’s area has a crucial role in motor control of speech. As of today, there is, however, no experimental evidence showing that this area has a direct function in motor control, i.e., a motor output measurable in specific muscles occurs when this region is stimulated, a distinctive characteristic all cortical motor areas. In the last decades, different studies have raised some criticism on the supposed motor function of Broca’s area (Flinker et al., 2015; Duffau, 2017; Rao et al., 2017), supported by two observations: pure lesions of Broca’s area do not result, as would be expected, in production aphasia, but rather in a transitory, rapidly improving mutism (Mohr et al., 1978); moreover the detailed analysis of the preserved brains of Broca’s patients clearly show that the lesion was not confined to BA44-45, but involved other structures beyond the frontal operculum (Dronkers et al., 2007), challenging the univocal correlation between production aphasia and a lesion of Broca’s area. The issue remains unresolved, due to the lack of appropriate experimental tools to study this area in humans in ecological conditions.

In the last two decades, the introduction of the intraoperative brain mapping technique for surgical removal of brain tumors with Direct Electrical Stimulation (DES) allowed the direct investigation of the functional properties of Broca’s area in ecologic conditions. The brain mapping technique relies on the premise that DES, delivered at the cortical and subcortical level in awake patients performing a behavioral task, interferes with its execution only when applied on structures belonging to the neural circuit sub-serving the ongoing task (Duffau and Duchatelet, 2016). In this setting, analysis of the deficit induced by DES provides elements relevant to disclosing the role of the stimulated area/fibers in the neural control of the task. The intraoperative stimulation of Broca’s area during speech production induces a so-called “speech arrest*”* (Luders et al., 1987; Axelson et al., 2009; Chang et al., 2011; Mandonnet et al., 2016; Chang et al., 2017), a well-known phenomenon in the neurosurgical literature, defined as “the complete interruption of ongoing speech in absence of oro-facial movements and vocal output” (Tate et al., 2014, 2015; Chang et al., 2017). This evidence supports the notion that Broca’s area has a critical role in speech production, but does not reveal precisely whether intraoperative stimulation of Broca’s area interferes with preceding semantic or phonological control necessary for speech production (for a review see Hickok, 2012), or whether it interferes directly with control of motor structures that produce speech. In a recent intraoperative study, we stimulated Broca’s area with both standard low frequency DES to induce “speech arrest,” and high frequency DES to induce motor evoked potentials (MEPs) in phono-articulatory muscles (Cerri et al., 2015). Stimulation of Broca’s area with short trains of high frequency DES failed to elicit MEPs in the hand or in oro-facial muscles, including phono-articulatory muscles active in speech, irrespective of muscle excitability (resting or pre-activated muscles) and/or functional state (during language tasks). The same paradigm applied to motor cortices (ventral pre-motor and primary motor cortex) elicited MEPs in both oro-facial and hand muscles. This result challenged the inclusion of Broca’s area among the well-established motor cortices hosting muscle representations. In the same study, low frequency DES induced “speech arrest” as expected, but careful observation suggested that it did so by aborting the onset, rather than “disrupting ongoing speech production.” This observation supports the hypothesis that the “speech arrest” induced by low frequency DES on Broca’s area, that is described by some neurosurgeons, may not be due to an impairment in execution of motor programs controlling the recruitment of phono-articulatory muscles’ motor units, but rather to an impairment in the pre-articulatory phase. This hypothesis, which in our previous study was based on descriptive comparison of EMG patterns of muscle activation, without and during DES delivered on Broca’s area, needs to be confirmed with a quantitative approach. Addressing whether “speech arrest” might in fact not be due to an arrest of ongoing muscle activity during speech is mandatory in order to challenge the involvement of Broca’s area in motor control of speech. Ultimate proof requires evidence of a functional connection of this area with motoneurons driving phono-articulatory muscles.

On the “cognitive side,” detailed evidence of the involvement of Broca’s area in pre-articulatory aspects of language comes from linguistic studies. The syllabification function, needed for the phonological encoding of words in sequential sounds, correlates with the activation of the entire Broca’s area (Indefrey and Levelt, 2004; Hickok, 2012). Moreover, semantic processing (Henry and Crawford, 2004; Costafreda et al., 2006; Robinson et al., 2012; Katzev et al., 2013; Bourguignon, 2014), required in single words as well as during composition of entire sentences (Hagoort, 2005; Vigneau et al., 2006; Price, 2010; Friederici, 2011), is attributed to the anterior-ventral sector (BA45) of Broca’s area, while phonological processing (Duffau et al., 2002; Costafreda et al., 2006; Katzev et al., 2013) and syntax processing (Vigneau et al., 2006; Price, 2010; Friederici, 2011) are attributed to the posterior-dorsal sector (BA44). Particularly interesting is the “map of speech sounds” (i.e., a topographic representation of frequent syllables) described in the ventral sector of BA44 (Guenther et al., 2006): the activation of this area is related to the phonetic translation preceding speech articulation (Long et al., 2016) in single word tasks, posing this area as the “phonetic encoder” of words (Hickok and Poeppel, 2004, 2007; Papoutsi et al., 2009).

In sum, when considering the literature investigating the anatomo-functional properties of Broca’s area, there are no doubts about its involvement in language, however, the precise nature of its role remains obscure, particularly in motor control of speech. The present study is the first to investigate the effect of intraoperative DES of Broca’s area on the activity of phono-articulatory muscles during single word tasks (object picture naming and counting), with a quantitative approach. The electromyography activity (EMG) of a sample of phono-articulatory muscles was recorded in 20 patients performing counting and naming tasks during DES on Broca’s area and analyzed in the frequency (power spectrum) and in the time (root mean square) domains. Should Broca’s area be critically involved in the control of motor output during speech, its stimulation during task performance is expected to affect the ongoing motor unit recruitment in the muscles involved in the task, and this effect is expected to be time-locked to the duration of the DES. Taking the analysis a step further, we also investigated whether the effect of DES can be attributed to a specific sector of the frontal operculum. To this aim, the topographic map of the responsive sites on Broca’s area was created and matched with the probabilistic map of terminations within the frontal lobe, of the main systems of fibers sub-serving the language network, reconstructed with High-Angular Resolution Diffusion Imaging (HARDI) q-ball tractography.

## Materials and Methods

The study was performed on 20 patients affected by gliomas during the surgical removal of tumor with the aid of the brain mapping technique. The aim of the study was to assess the actual involvement of Broca’s area (BA44-45) in motor control of speech. To this aim we focused the investigation on the EMG activity of the phono-articulatory muscles active during two speech tasks (object picture naming and counting) performed by patients during awake surgery for brain tumor removal in two conditions, i.e., during and in absence of DES stimulation of Broca’s area. The EMG of each muscle recorded during task performance in the two conditions was analyzed offline in frequency (power spectrum, PS) and time domain (root mean square, RMS) and the two conditions compared.

A comprehensive summary of methods is provided in Box 1. A detailed description of methods reports, below, the inclusion criteria, the surgical procedure, the intraoperative brain mapping technique, the EMG and neuroimaging data analysis.

### Inclusion Criteria and Surgical Procedure

#### Patient Selection

Twenty patients affected by gliomas were enrolled in this study. All the patients had left language dominance. Tumors were localized in the left frontal, temporal and/or insular lobes, never infiltrating or reorganizing Broca’s area (see for inclusion criteria Fornia et al., 2016). All patients were free from neurological deficits affecting the motor and/or language functions (see **Table 1** for detailed description of all patients). All patients gave written informed consent to the surgical and mapping procedure, which followed the principles outlined in “World Medical Association Declaration of Helsinki: Research involving human subjects.” The study was performed with strict adherence to the routine procedure normally utilized for surgical tumor removal. Accordingly, all data were recorded utilizing electrophysiological monitoring and stimulating protocols (see below) adopted for routine clinical mapping.

**Table 1:** Clinical classification of patients undergoing the analysis.

ID patients	Age	Lesion site	Lesion side	Tumor histology	Grade (WHO)	Clinical history		AEDs number
						Motor deficits	Phono-articulatory deficits	
Patient 01	56–60	T	L	Glioblastoma	IV	–	–	1
Patient 02	20–25	F	L	Anaplastic oligodendroglioma	III	–	–	2
Patient 03	20–25	FI	L	Glioblastoma	IV	–	–	1
Patient 04	46–50	FI	L	Oligodendroglioma	II	–	–	3
Patient 05	30–35	FI	L	Oligoastrocytoma	II	–	–	2
Patient 06	46–50	TI	L	Anaplastic oligoastrocytoma	III	–	–	2
Patient 07	20–25	FI	L	Oligoastrocytoma	II	–	–	1
Patient 08	40–45	T	L	Metastasis		–	–	1
Patient 09	46–50	F	L	Anaplastic oligodendroglioma	III	–	–	1
Patient 10	40–45	TI	L	Oligodendroglioma	II	–	–	2
Patient 11	36–40	TI	L	Anaplastic astrocytoma	III	–	–	1
Patient 12	36–40	FI	L	Anaplastic astrocytoma	III	–	–	1
Patient 13	40–45	FI	L	Oligoastrocytoma	II	–	–	1
Patient 14	40–45	F	L	Oligodendroglioma	II	–	–	1
Patient 15	36–40	T	L	Oligodendroglioma	II	–	–	1
Patient 16	40–45	T	L	Glioblastoma	IV	–	–	1
Patient 17	16–20	TI	L	Astrocytoma	II	–	–	1
Patient 18	56–60	T	L	Cavernous angioma		–	–	0
Patient 19	26–30	F	L	Oligodendroglioma	II	–	–	3
Patient 20	30–35	T	L	Astrocytoma	III	–	–	1

*Lesion: F, Frontal; T, Temporal; FI, Fronto-Insular; TI, Temporo-Insular; AEDs, Anti-Epileptic Drugs.*

#### Pre-operative Routine

In the pre-operative routine assessment, all patients were submitted to handedness assessment, neurological examination and a neuropsychological evaluation of cognitive abilities as the non-verbal intelligence, memory, praxis and language. The neuroradiological examination included morphological T1, T2, FLAIR, DWI and post contrast T1 images (Bello et al., 2014). A functional MRI (fMRI) study was performed in all patients to localize three neighboring cortical areas of the frontal lobe: (i) the primary motor cortex (M1), localized with a finger-tapping task, (ii) the Broca’s area and (iii) the ventral pre-motor cortex (vPM) identified with a covert visual naming and fluency task or covert auditory verb generation (Ruge et al., 1999; Cerri et al., 2015). The language hemispheric dominance was determined by the laterality index, based on the fMRI results in both language tasks. Following the fMRI investigation, High-Angular Resolution Diffusion Imaging (HARDI) q-ball (6 patients) or Diffusion Tensor Imaging (DTI) (14 patients) tractography reconstructions allowed for reconstruction and visualization of the motor and/or language fibers in the frontal lobe to estimate their anatomical relationship with the lesion.

All the data acquired in the pre-operative routine assessment contributed to the design of the optimal functional mapping strategy to be performed during the surgical tumors removal.

#### Surgical Procedure and Intraoperative Routine

Patients were subjected to an asleep-awake-asleep anesthesia. The surgery was performed with the aid of the brain mapping technique for cortical and subcortical mapping. According to this technique, DES was applied on cortical areas to define the surgical cortical entry zone, while subcortical mapping was performed along with tumor resection to locate functional motor and/or language fibers representing the limit of resection (Bello et al., 2014).

##### Neurophysiological brain monitoring

The cortical activity was monitored, during the procedure, by ElectroEncephaloGraphy (EEG, Comet) and ElectroCorticoGraphy (ECoG, Grass). ECoG was recorded from a cortical region adjacent the area to be stimulated by subdural strip electrodes (4–8 contacts, monopolar array referred to a midfrontal electrode) through the whole procedure, to monitor the basal cortical electrical activity and to detect after-discharges or electrical seizures during the resection. EEG was recorded with electrodes placed over the scalp in a standard array. EEG and ECoG signals were filtered (bandpass 1–100 Hz), displayed with high sensitivity (50–150 μV/cm and 300–500 μV/cm respectively) and recorded.

The integrity of the essential descending motor pathways was monitored throughout the procedure using the so-called “train-of-five” (To5) monitoring technique. Trains of 5 stimuli were delivered from the beginning to the end of the procedure to M1 cortex to elicit Motor Evoked Potentials (MEPs) in contralateral oro-facial and hand muscles in order to monitor the integrity of the corticospinal transmission (for details see Bello et al., 2014).

During surgery, the muscle activity of the patient was recorded by pairs of subdermal hook needle electrodes (Technomed) inserted into 20 muscles (face, upper and lower limb) contralateral to the hemisphere to be stimulated, plus 4 ipsilateral muscles connected to a multichannel EMG recording (ISIS, INOMED, sampling rate 20 kHz, notch filter at 50 Hz) (Bello et al., 2014). EMG was used to record the responses to stimulation, either the To5 MEPs responses or the responses elicited during the brain mapping, the voluntary motor activity and to distinguish between electrical and clinical seizures. Close attention was paid to prevent intraoperative seizures, with the ECoG and EMG monitoring: as clinical routine procedure at the first ictal sign, the stimulation was stopped and cold irrigation was applied, to abort the seizure and whenever the seizures spread to the whole hemibody, propofol bolus infusion (4 ml on average) was delivered.

##### Neurophysiological brain mapping

The brain mapping technique for cortical and subcortical mapping of both motor and language components can be performed with two different stimulation paradigms: Low Frequency (LF) and High Frequency (HF) stimulation (Bello et al., 2014).

In the present study, we focused the analysis of EMG data obtained during brain mapping performed with the LF stimulation (LF-DES) delivered on Broca’s area while the patients were performing two speech tasks, i.e., the object picture naming and the counting task (see below). According to the clinical procedure, both tasks were performed in two different conditions: in absence of DES stimulation (DES-OFF condition) and during LF-DES delivered on Broca’s area (DES-ON condition). The LF stimulation consisted in trains (1–4 s duration) of biphasic square wave pulses (0.5 ms each phase) at 60 Hz (ISI 16.6 ms) delivered by a constant current stimulator (OSIRIS-NeuroStimulator) integrated into the ISIS-System through a bipolar probe (two ball tips, 2 mm diameter, spaced by 5 mm).

*Intraoperative speech tasks.* Object picture naming task: the patient was asked to name the picture of objects randomly presented on a computer screen. Pictures were presented with a regular timing allowing a pause of few seconds between subsequent pictures. As soon as the picture was presented, the patient was asked to name the picture, while during the pauses the patient was silent (resting).

Counting task: the patient was asked to count from one to ten. The count was self-paced, but patients were previously trained to wait few seconds (resting) between one number and the following one.

During task performance (either counting or object picture naming), LF-DES was randomly applied on the same stimulation site, so that some trials were performed without stimulation (DES-OFF) and others during stimulation (DES-ON). When the naming task was performed during stimulation, the surgeon delivered DES at the presentation of the object to be named. DES during counting task was delivered in the pause between to subsequent numbers to be pronounced. No external cues were given to the patients to pace the onset of the speech.

*Intraoperative identification and stimulation of Broca’s area.* According to clinical procedure, for each patient the pre-operative anatomo-functional identification of Broca’s area, to be distinguished from the ventral pre-motor area (vPM) and the primary motor cortex (M1), was performed with a dedicated neuroimaging (fMRI-DTI) study (Ruge et al., 1999; Cerri et al., 2015). The fMRI and DTI or HARDI data were loaded on the neuronavigation system to be available for the neurosurgeon. During the procedure, the conclusive identification of the three areas to define the point of surgical entry was mandatory and performed with the brain mapping technique. LF stimulation delivered in resting condition is highly effective on M1 inducing overt motor responses (orofacial movements), clearly recorded by the EMG electrodes, while it is not effective when applied to vPM and Broca’s area. Conversely, LF-DES delivered on the three areas during speech tasks, impairs the task performance although with different clinical features, thus allowing the surgeons to distinguishing among the different cortices. The occurrence of speech disturbances upon application of DES to the three areas, measured by clinical inspection, has been indeed extensively documented (Matsuda et al., 2014; Tate et al., 2014; Chang et al., 2017) and, therefore, is part of the clinical routine in functional neurosurgical practice. As a standard routine, when the stimulation of Broca’s area stops (“speech arrest” phenomenon) without inducing movements, the patient’s counting/naming at least three non-consecutive times, the identification of Broca’s area is considered reliable. Regarding vPM, when the stimulation induces disruption of speech referred as “anarthria” (a term so far considered a synonymous of speech arrest) (Tate et al., 2014, 2015), at least three non-consecutive times, the identification of the area is considered reliable. Differently, the stimulation of M1 induces a disruption of speech with facial muscles contraction (Tate et al., 2014) and referred as “dysarthria” (a motor impairment accompanied with dysphonic/aphonic speech) (Deletis et al., 2014).

According to the routine procedure for language mapping, in our patients the first areas to be identified were M1 and/or vPM. LF-DES paradigm was applied on M1 (on face motor areas) and/or vPM to identify the minimum current intensity (Threshold Intensity) needed to induce a clear interference (dysarthria and/or anarthria/speech arrest) in task performance (*ThreshI*-DES). Then DES was applied onto the putative Broca’s area and the intensity of stimulation was initially set at *ThreshI*-DES and then increased until the “speech arrest” was obtained (*SupraThreshI*-DES). This protocol was applied only when it was clinically mandatory for the surgeon to define a clear border between Broca’s area and the neighboring vPM. In our study, the *SupraThreshI*-DES was applied only in 7 out of 20 patients.

Considering all the patients enrolled, the main parameters of LF-DES protocol applied on the three areas were:

(i) Broca’s area: average train duration ± SD: 2.8 ± 1.0 s; average stimulation intensity ± SD: 3.4 ± 1.3 mA;
(ii) vPM: average train duration ± SD: 2.2 ± 1.0 s; average stimulation intensity ± SD:2.7 ± 0.7 mA;
(iii) M1: average train duration ± SD: 2.1 ± 0.8 s; average stimulation intensity ± SD: 2.7 ± 1.0 mA.

The complex clinical setting and the primary concern to avoid any impact on the clinical procedure, did not allow recording the same number of trials in each patient.

In each patient, the EMG activity of the entire set of muscles was recorded during the cortical mapping. A dedicated channel, recording the patient’s vocal production, was simultaneously recorded. In particular, for the offline analysis we focused on responses of a sample of phono-articulatory muscles: superior *orbicularis oris, mylohyoid, mentalis* contra- and ipsilateral to the left hemisphere and the contralateral *platysma*.

### EMG Analysis

The EMG of the phono-articulatory muscles during speech tasks performance was recorded in two conditions: in absence of stimulation (DES-OFF) and during stimulation (DES-ON). Offline analysis of intraoperative data was performed at single subject level as follows:

(1) Selection of the EMG activity occurring in phono-articulatory muscles during task performance in the two conditions: DES-OFF and DES-ON.
(2) Quantitative characterization, in frequency and time domain, of the pattern of EMG activity recorded in phono-articulatory muscles during DES-OFF and during DES-ON.
(3) Statistical comparison of pattern of EMG activation in the two conditions.

#### Selection of the EMG Activity Occurring in Phono-Articulatory Muscles during Task Performance in the Two Conditions: DES-OFF and DES-ON

The first analysis was aimed at extracting from the EMG recorded at baseline, the EMG signal corresponding to the task performance both in absence of stimulation and during stimulation. To this aim the EMG signal recorded in both DES-OFF and DES-ON conditions was analyzed, offline, using a dedicated software (MatLab) allowing to distinguishing and extracting from the baseline EMG, the EMG signal corresponding to the task performance. The same data analysis was performed separately for the object picture naming and counting tasks.

**FIGURE 1:**
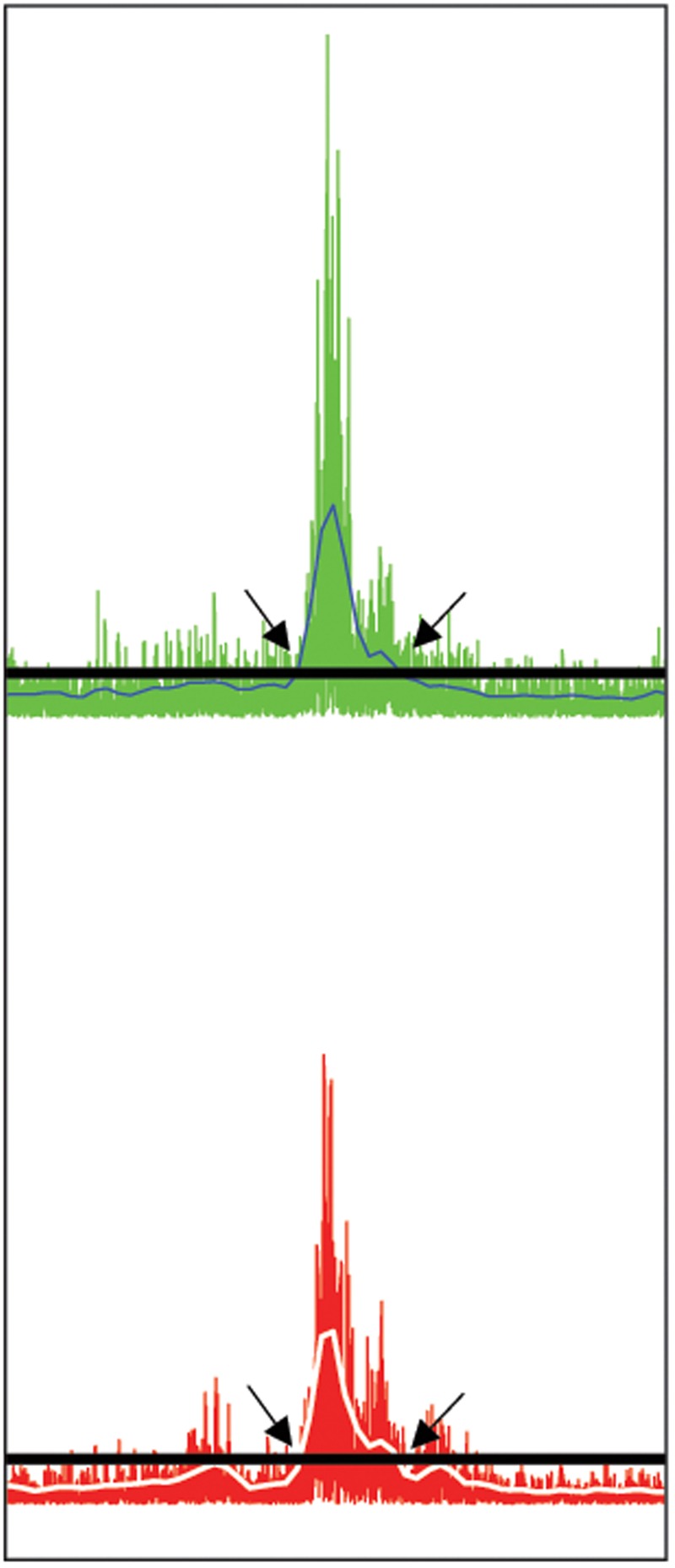
Selection of phono-articulatory muscles activity. Illustration of the methods used for the extraction of the EMG segments to be entered in the RMS and PS analysis. The EMG segment was extracted at the ONSET and OFFSET of the EMG activation based on the RMS calculation (see Materials and Methods). The extracted segment was then processed for both RMS and PS activity. Exemplary illustration from the EMG signal related to *Orbicularis Oris* contra- **(upper trace)** and ipsilateral **(lower trace)** muscle. Both EMG signals are rectified. The horizontal lines (black) correspond to the value of three times the standard deviation (3^∗^SD) of the mean amplitude (in μV) of the baseline EMG, while the white lines on EMG signals correspond to Root-Mean-Square (RMS) blue line in upper trace and white line in lower trace. The onset of the EMG activity related to DES-OFF was set at the point of intersection between the 3^∗^SD line and the increase of RMS slope (arrows at left). Accordingly, the offset of the EMG activation during tasks was set at the point of intersection between the line and the decrease of RMS slope under the 3^∗^SD line (arrows at right).

Two subsequent analysis were performed: (A) the EMG signal corresponding to the muscle activation occurring during DES-OFF was selected with respect to the EMG signal corresponding the resting condition (baseline); (B) the EMG signal corresponding to muscle activation occurring during DES-ON was selected based on the stimulus artifact, recorded in a dedicated EMG channel, indicating the exact time window of the stimulus;

(A) DES-OFF condition. To select and extract the EMG segment corresponding to the muscle activation occurring during the utterances pronounced in DES-OFF condition, the EMG of each phono-articulatory recorded muscle was rectified and the Root-Mean-Square (RMS) calculated across an epoch (time window) of 100 ms, with a sliding window of 50 ms to the preceding one. RMS of the baseline activity was calculated by averaging four fragments of EMG signal in resting condition selected randomly (length of each part about 2 s) plus its 3^∗^SD. The latter value was chosen to exclude from the baseline signal the non-specific muscular activity occurring during small and non-task related movements, possibly creating a false positive EMG activation. For each utterance (trial), the onset and the offset of the task-related muscle activity were extracted by subtracting the RMS baseline activity from each trial of the task, i.e., by setting at the point of intersection between the 3^∗^SD line and the RMS slope corresponding to the onset or offset of the EMG activation of the phono-articulatory muscle during tasks. Illustration of the methods used for the extraction of the EMG segments to be entered in the RMS and PS analysis is presented in **Figure 1**. The duration of the EMG segment corresponded to the time needed to produce the utterance, which was normally short and variable among patients. All the EMG segments corresponding to all trials in DES-OFF conditions were entered in the quantitative EMG analysis (see Quantitative Characterization of the Pattern of EMG Activity Recorded in Phono-Articulatory Muscles during DES-OFF and during DES-ON Condition).

A total of 369 (considering all patients), trials were recorded in DES-OFF condition, irrespective of the speech task. Number of DES-OFF trials per patient ranged 4–47 (Naming: mean 18 trials/patient ± 9SD; Counting: mean 11 trials/patient ± 14 SD).

(B) DES-ON condition. The EMG signal corresponding to DES-ON condition (either *TreshI-DES* and *SupraThreshI-DES*) was selected by using as a reference the stimulation artifact recorded by one of the EMG synchronized-channels (*Orbicularis Oculi*). The EMG segment selected (trial) corresponded to the onset and offset of the stimulation and its duration corresponded to the duration of the stimulus artifact. All the EMG segments corresponding to all trials in DES-ON conditions were entered in the quantitative EMG analysis (see Quantitative Characterization of the Pattern of EMG Activity Recorded in Phono-Articulatory Muscles during DES-OFF and during DES-ON Condition).

A total of 97 trials, considering all patients, were recorded in DES-ON condition, irrespective of the speech task (mean number of trials for subject ± SD = 5 ± 4). Within the 97 trials are included both stimulation failing to elicit any effect (DES-ON *ThreshI-DES*) and stimulation inducing speech prevention (DES-ON *SupraThreshI-DES*). Number of DES-ON trials per site ranged 3–10.

All recorded-data (369 DES-OFF and 97 DES-ON) entered the statistical analysis (performed at the single patient level) in both time and frequency domain parameters. The higher number of DES-OFF trials and its variability among patients were due to the clinical requirements, which were different in different procedures. In some patients the tumor was located near (but not infiltrating) to cortical and subcortical areas involved in language and other cognitive/neurological functions, while in other it was embedded within the areas and pathways related to language and speech (not infiltrating). In the latter patients the brain mapping procedure with speech tasks needed to define the functional borders of the tumor was performed more extensively with respect to the former patients and the number of trials was high. For this reason, a wide range of DES-OFF trials per patient (4–47) was collected. For the same reason, when the mapping was focused at disclosing the border between Broca’s area and the neighboring cortex, the number of DES-ON trials per patient was lower ranging 3–10 trial per patient.

#### Quantitative Characterization of the Pattern of EMG Activity Recorded in Phono-Articulatory Muscles during DES-OFF and during DES-ON Condition

The EMG segments selected with the previous analysis (DES-ON condition: 97 trials and DES-OFF condition: 369 trials) was processed with a quantitative analysis in order to obtain the distinguishing features characterizing the muscle activation in the two conditions. To this aim the EMG segments of the recorded muscles was analyzed, in both conditions, in frequency (power spectrum, PS) and in time (root mean square, RMS) domain. This approach allows for the quantitative characterization of the motor units recruitment in the different muscles active in the two conditions. The same analysis was applied to each muscle to characterize also the EMG activity in baseline, i.e., when muscles were not active in performing speech (e.g., EMG recorded in the pause within two words to be produced). This analysis was mandatory to correctly identify the onset of the EMG activity during task performance.

##### Analysis in time domain

The RMS was selected as the main parameter for the analysis of the EMG signal in time domain: the mean and peak values (in μV) RMS were compared in the two conditions.

##### Analysis in frequency domain

The PS of the signal (Fast Fourier Transform) was computed to estimate the mean and median frequency and the area (in μV^2^) under the spectrum curve. Notably, in each power spectrum lacks the information related to power of 50 Hz signal, since a safety notch filter (at 50 Hz) is applied by machine (ISIS, INOMED) to exclude alternate current by signals.

The statistical analysis was performed at the single subject level, not at the population level. **Supplementary Figure S1** reports the EMG quantitative analysis values calculated in a single subject. For each muscle, the EMG parameters were calculated in frequency and time domain in the four different experimental conditions reported: DES-ON (*SupraThreshI-DES*)/Speech prevention, Baseline, DES-OFF and DES-ON (*ThreshI-DES*)/No effect.

In all patients and in all conditions the EMG analysis was always matched with the functional outcome, i.e., the clinical evaluation of the task performance routinely assessed by the neuropsychologists during the procedure as part of the clinical procedure.

#### Statistical Comparison of Pattern of EMG Activation in the Two Conditions

For each patient, a statistical analysis was applied to compare the two conditions: DES-OFF and DES-ON. The aim was to disclose whether DES applied on Broca’s area during task performance actually exerts a disruption of the ongoing motor program interfering with the physiological motor unit recruitment occurring during DES-OFF, used as main reference. For each muscle analyzed, the comparison of the EMG activity recorded during DES-OFF (369 trials) and during DES-ON condition (97 trials) was computed on all the five parameters calculated: the mean and peak values for RMS and the mean and median frequencies, and area under the curve for PS. Statistical analysis was performed in Statistica 7.1 software, by means of Mann–Whitney *U*-test. The EMG analysis was conducted for each single patient separately. Since we compared the effect of DES on Broca’s area during speech (DES-ON) vs. speech without DES (DES-OFF) as a main reference, we treated the two conditions as independent, therefore a test for independent samples was chosen. The non-parametric test for independent samples, particularly the Mann–Whitney *U*-test, was adopted since a small sample of trials were available for a single patient and the distribution tests lack of sufficient power to provide meaningful results. The significance level adopted was *p* < 0.05. The analysis of the EMG during object picture naming and counting tasks was performed separately.

### Neuroimaging Analysis

Postoperative neuroimaging analysis was performed to allow the precise localization of the DES-induced effect on Broca’s area at single subject and population level, and to estimate the putative correlation of the effect obtained by stimulating Broca’s cortex and the main systems of connecting fibers involved in language.

#### 3D Map of the LF-DES Stimulation Sites on Broca’s Area

For each patient, the reconstruction of the exact position of the sites stimulated with both *ThreshI*-DES and *SupraThreshI*-DES on Broca’s area and on the neighboring vPM/BA6 was computed. During the intraoperative mapping, the coordinated of the stimulation sites effective in inducing the impairment of the task were recorded on the neuronavigator system. To report the exact position of the sites on the 3D MRI surface of the patients, the following procedure was adopted. The MRI-T1 volume of each patient was used to perform the cortical surface extraction and the surface volume registration was computed with the Brainsuite (Shattuck and Leahy, 2002) dedicated software. Data were then loaded into Brainstorm (MatLab Tool Box^1^) (Tadel et al., 2011) and the exact position of the coordinates was labeled onto the 3D patient’s MRI. Subsequently the 3D MRI and the labeled points were co-registered to the MNI space system (non-linear ICBM 152). This procedure allowed to report each recorded stimulation point into the MNI coordinates space system (Fornia et al., 2016). Coordinates of each point were then labeled onto the ICBM 152 to create a 3D reconstruction of the left stimulated hemisphere. The Unified segmentation (Ashburner and Friston, 2005) was used to normalize each brain and the related stimulated sites to the MNI space. However, since this transformation may introduce significant spatial location inaccuracies, we visually inspected the location of the stimulated sites on the MNI template and matched it with the original coordinates of patient’s brain. All the sites in BA44/45 and ventral portion of the precentral gyrus located on the MNI template matched with the sites originally identified on patient’s brain. This procedure reduced at best the inaccuracies of the transformation.

#### MR Tractography Analysis

By means of High-Angular Resolution Diffusion Imaging (HARDI) q-ball tractography technique, the cortical terminations of the main white matter bundles related to the language function were reconstructed to investigate whether they reach the sites of stimulation on Broca’s area and therefore might be involved in the genesis of the effect observed and characterized with the quantitative analysis.

MR Tractography was performed on six out of the seven patients that received *SupraThresholdI-DES*. HARDI datasets were collected in 6 right-handed patients on a 3T scanner (3T Achieva, Philips Healthcare) using a diffusion-weighted spin echo EPI single-shot pulse sequence with the following parameters: 60 diffusion gradient directions, *b*-value = 3000 s/mm^2^, TR/TE = 12000/74 ms, SENSE factor = 2, in-plane resolution = 1.87 × 1.87 mm^2^; thickness = 2.5 mm; no gap, FOV = 240 mm^2^, acquisition matrix = 128 × 128, data averages = 1, total scan time 13 min. A 3D T1-weighted sequence and a 3D-FLAIR sequence were also acquired for anatomical characterization.

HARDI datasets were corrected for movement and eddy-current distortions using FMRIB Software Library (FSL). Diffusion Imaging in Python (Dipy) software was used to estimate fractional anisotropy (FA) and for *q*-ball residual-bootstrap fiber tracking of language pathways (Caverzasi et al., 2014, 2016). Tracking was performed using an FA threshold = 0.1 and max angle = 60° as stopping parameters in the algorithm. Tractography of language pathways was performed by a board-certified neuroradiologist (A.C., with 11 years of experience in MR tractography analysis). The main white matter bundles belonging to language pathways (from Chang et al., 2015; Kinoshita et al., 2015) and having terminations in the frontal lobe (arcuate fasciculus [AF], superior longitudinal fasciculus component II [SLF-II], SLF component III [SLF-III], SLF component temporo-parietal [SLF-tp], inferior fronto-occipital fasciculus [IFOF], uncinate fasciculus [UF] and frontal aslant tract [FAT]) were reconstructed using a 2- or 3-ROI approach, depending on the bundle (Caverzasi et al., 2016). Results were visualized using Trackvis^2^. Specifically, to reconstruct the IFOF and UF a single-plane seed ROI was defined on the FA color map in the coronal plane passing through the anterior commissure, by selecting the anterior part of the left external and extreme capsules, where the two tracts run in contiguity. Target ROIs for the UF and IFOF were localized at the levels of the temporal and occipital lobes, respectively. For both tracts, the left frontal lobe was used as a second target ROI. Streamlines that passed through both target ROIs were retained. To reconstruct SLF-II and -III and AF a seed ROI was positioned in the coronal plane at the level of a region of high anteroposterior anisotropy lateral to the central part of the lateral ventricle and the corona radiata. Target ROIs were selected as follows: in the angular gyrus for SLF-II, in the supramarginal gyrus for SLF-III, and on the axial peritrigonal plane at the level of the posterior middle and superior temporal gyri for the AF. The left frontal lobe was used as a second target ROI. Streamlines that passed through both target ROIs were retained. To reconstruct FAT, the first region of interest was located in the white matter of the inferior frontal gyrus and the second region of interest in the white matter of the superior frontal gyrus, including the anterior cingulate and pre-supplementary motor area (Catani et al., 2013).

For each patient, the FA maps were co-registered to the anatomical images and to the FSL 2 mm × 2 mm × 2 mm resolution Montreal Neurological Institute (MNI) atlas using FSL linear and non-linear transformations (FMRIB’s FLIRT and FNIRT registration tools). Density maps of the end points of each fiber tract were saved in the patients’ native space using TrackVis and thresholded to obtain binary masks containing all voxels that were visited by at least one streamline in the *q*-ball residual-bootstrap tractography. These masks were spatially normalized to the MNI space using the linear and non-linear transformations derived from the co-registration of the FA maps to the FSL MNI atlas. All patients’ end point maps in the MNI space for each tract were summed to visualize the distribution of the tract terminations and their overlap with intraoperative stimulation sites normalized to the MNI space. A 3D rendering of the end points of the different tracts was obtained using the FSL 3D viewer.

## Results

The involvement of Broca’s area in motor control of speech was investigated during the surgical removal of brain tumors, performed with the aid of the brain mapping technique. The main focus of the study was the investigation of a functional connection of Broca’s area with motoneurons driving phono-articulatory muscles. To this aim we investigated whether the intraoperative stimulation of Broca’ area affects the motor unit recruitment of the phono-articulatory muscles active during speech, in 20 patients. For each patient two different intraoperative conditions were compared: (i) the DES-OFF: speech production in absence of stimulation, and (ii) the DES-ON: speech production during direct low-frequency current stimulation applied onto Broca’s area during speech tasks. Muscle activity during object picture naming and counting task was investigated with a quantitative analysis in both frequency and time domain, to evaluate which domain reveals the distinguishing features of the recruitment of motor units in the two conditions. In all patients and all conditions, the EMG analysis was always matched with the functional outcome, i.e., the clinical evaluation of the task performance routinely assessed by the neuropsychologists during the procedure as part of the clinical procedure.

Main Results can be summarized as follows:

(1) DES on Broca’s area induces an intensity-dependent “speech arrest”: the intensity of DES needed to induce “speech arrest” when applied on Broca’s area was indeed higher when compared to the intensity effective in inducing speech impairments when delivered on the neighboring pre-motor/motor cortices.
(2) The quantitative parameters measured in frequency (PS) and time (RMS) domain on EMG recorded during “speech arrest” were comparable to those measured on the EMG recorded at baseline as if the motor program never started.
(3) Speech arrest induced by DES on Broca’s area was an “all-or-none” effect: partial interruptions of speech were not observed. Muscle activation started only by removing DES, as if DES prevented speech onset.
(4) DES on Broca’s area never affects the motor unit recruitment in either naming or counting tasks, coherently with the functional outcome reporting a lack of any deficit in phono-articulation.
(5) Speech arrest is an effect obtained when stimulating a specific sector of Broca’s area, i.e., the ventral BA44.
(6) No semantic or phonological deficits were observed when stimulating BA44-45 with the speech tasks adopted in this study.
(7) Speech arrest with the same features was observed also when stimulating directly the subcortical fibers running below Broca’s area.

### Effect of LF-DES Applied on Broca’s Area during Speech Tasks: Functional Outcome vs. EMG Analysis

EMG quantitative analysis and the functional outcome show that the effect of Broca’s area stimulation during speech is an all-or-none effect strictly dependent of the intensity of stimulation.

According to clinical procedure, for each stimulation site on Broca’s area the intensity of LF-DES was initially set at Threshold Intensity (*ThreshI*-DES), i.e., the minimum intensity inducing a disruption of ongoing speech when applied on M1 and/or on vPM (see Materials and Methods). The average *ThreshI-*DES value (average stimulation intensity ± SD) applied on Broca’s area was 2.7 ± 0.7 mA (train duration of 2.8 ± 1.0 s). Each site was stimulated for a minimum of three non-consecutive times. DES-ON stimulation trials were alternated with DES-OFF trials. The effect of stimulation with *ThreshI-*DES on task performance was evaluated by clinical inspection (vocal outcome) during surgery and by the quantitative offline analysis of the EMG activity (see Materials and Methods). All values were then compared with those measured during DES-OFF. Due to clinical needs, in 7 out of 20 patients the intensity of LF-DES was increased (*SupraThreshI-*DES). Again, the effect of stimulation with *SupraThreshI-*DES on task performance was evaluated by clinical inspection and by the quantitative offline analysis of the EMG activity and all values were compared with those measured during DES-OFF. Statistical differences (Intensity and Duration) between *SupraThreshI-DES* and *ThreshI*-DES in the population of 7 patients receiving both was assessed by means of Mann–Whitney *U*-test. Analysis showed that the DES failing to induce the effect (*ThreshI*-DES) and DES inducing the effect (*SupraThreshI*-DES) significantly differed in terms of Intensity (*U* = 130.5, *P* < 0.01, mean *SupraThreshI-*DES 4.3 ± 1.3 mA; mean *ThreshI-*DES 3.1 ± 1.3), but not in terms of Duration (*U* = 199.5, *P* = 0.07, mean *SupraThreshI-*DES 4.3 ± 1.3 mA; mean *ThreshI-*DES 3.1 ± 1.3) suggesting that “speech arrest” is intensity dependent effect.

The following paragraphs report in details the effect of *ThreshI-*DES and *SupraThreshI-*DES on functional performance and on the motor unit recruitment as assessed by the quantitative analysis of the EMG (see Materials and Methods).

#### Threshold LF-DES on Broca’s Area

When *ThreshI*-DES was delivered on the ventral sector of pre-motor cortex, vPM, i.e., the area located posterior to Broca’s area, it disrupted task performance by inducing a dysfunctional articulation of the word being pronounced and/or vowel emission. During stimulation of vPM, patients attempted to pronounce a word but phono-articulation was not appropriate, as shown by stuttering and stopping, and by the pattern of ongoing EMG of the active muscles, which is altered with respect to the pattern occurring in DES-OFF (Cerri et al., 2015).

**FIGURE 2:**
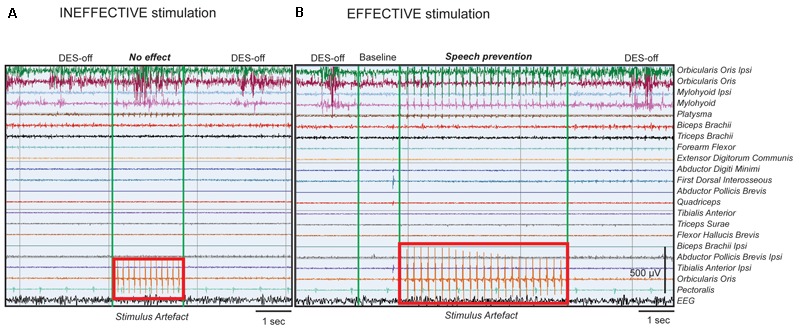
DES’s effect on Broca’s area during speech tasks. Example of intraoperative EMG recordings in a single patient, performing counting task. All muscles recorded in intraoperative setting are shown and indicated on the right. **(A)** The EMG trace represents, in sequence: left panel, the *natural* speech *performance* without stimulation (DES-OFF), middle panel, the stimulation applied onto Broca’s area with *ThreshI*-DES (4 mA, stimulus artifact represented in the red rectangle on muscle channel) and right panel, again DES-OFF. *ThreshI*-DES on Broca’s area does not seem to interfere with speech production. The EMG signal during DES appears similar to that of DES-OFF. **(B)** The EMG trace represents, in sequence: left panel, DES-OFF, second panel, baseline signal, third panel, stimulation applied onto Broca’s area with *SupraThreshI*-DES (6 mA, stimulus artifact represented in the red rectangle on muscle channel) and right panel, again DES-OFF. *SupraThreshI*-DES on Broca’s seems to *prevent* speech production. The EMG signal during DES appears similar to that of the baseline and not to that of DES-OFF. When the stimulus was applied onto the cortex, in cranial muscles’ channels (mainly phono-articulatory muscles, see first 4 channels) and in a dedicated channel (Orbicularis Oculi, red rectangle) the stimulus artifact was recorded. The pure artifact of stimulation recorded in the Orbicularis Oculi channel was used as reference time window to select the EMG of the phono-articulatory muscles during DES (see Materials and Methods). The stimulus artifact, visible in the figure in phono-articulatory muscles, was filtered to perform the EMG quantitative analysis. The signal was processed with different notch filters at 60 Hz and its harmonics.

**FIGURE 3:**
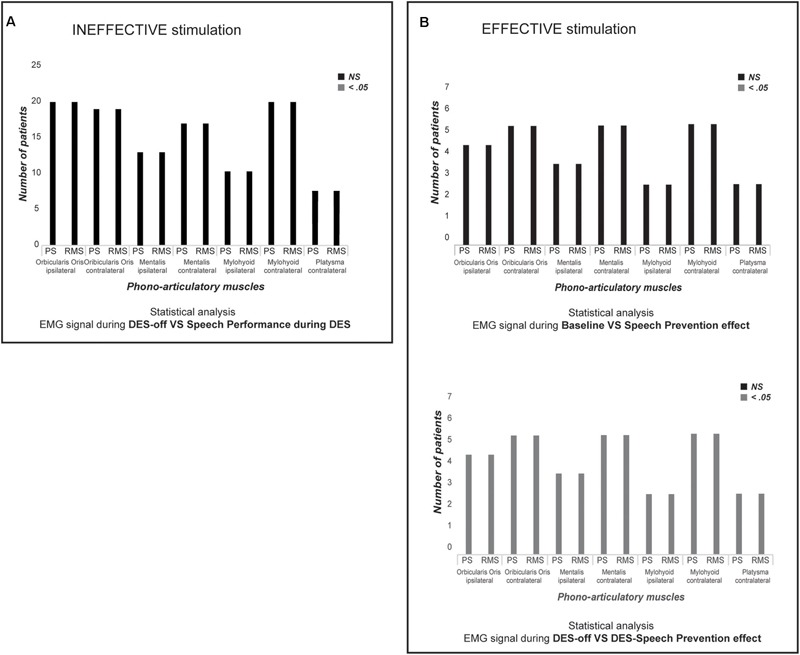
Statistical analysis. **(A)** The chart shows, for each recorded muscle in all twenty patients, if there is a significantly difference (Mann–Whitney *U*-test) between each PS and RMS parameters in DES-OFF vs. performance during *ThreshI*-DES. Black bars indicate that there is Not a Significantly (NS, *p* > 0.05) difference between two conditions. Instead, gray bar would indicate a significantly different (*p* < 0.05). *ThreshI*-DES on Broca’s area does not interfere with speech production. **(B)** The chart shows, for each recorded muscle in seven patients in which was applied *SupraThreshI*-DES, if there is a significantly difference (Mann–Whitney *U*-test) (upper chart) and between each PS and RMS parameters at DES-OFF vs. performance during *SupraThreshI*-DES (lower chart). Black bars indicate that there is Not a Significantly (NS) difference between two conditions. Instead, gray bar would indicate a significantly different (*p* < 0.05). *SupraThreshI*-DES on Broca’s *prevents* totally the speech production. The phono-articulatory recorded-muscles and the EMG calculated-parameters (PS and RMS) are indicated on *x*-axis. Only for the graphic representation, the PS parameters (mean and median frequency, and the area under PSs curve) and the RMS parameters (mean and peak) are grouped together. The number of analyzed-patients is indicated on y-axis. PS, Power Spectrum; RMS, Root Mean Square.

Conversely, when *ThreshI*-DES was delivered to Broca’s area just prior or during speech initiation, no effect was observed on speech production (anarthria/“speech arrest,” dysarthria or an interruption of ongoing speech), or on semantic and phonological processing (**Figure 2A**). During stimulation, all patients could speak normally and performed both tasks with precision. Quantitative analysis of the EMG activity showed, in all patients, no significant differences in muscle activation between DES-OFF and DES-ON (**Figure 3A** and **Supplementary Table S1A**): mean and median frequencies, and PS area were comparable in the two conditions (*p* > 0.05), as well as mean and peak RMS values (*p* > 0.05).

In summary, DES on Broca’s area, delivered at threshold intensity, as defined by its effectiveness on the vPM area, did not affect motor unit recruitment in either naming or counting tasks. This is coherent with the functional outcome, reporting a lack of any phono-articulatory deficits.

#### SupraThreshold LF-DES on Broca’s Area

In seven patients, the intensity of LF-DES was increased until an effect was observed (*SupraThreshI*-DES): the patients did not, and did not even try to, articulate the word/number. This effect is commonly referred to as “speech arrest.” Notably, inability to pronounce the word occurred without any evident attempt to do so, as if the stimulation prevented the onset of speech altogether, rather than blocking the muscles’ activity during speech (**Figure 2B**). As soon as the stimulus was removed, patients performed the task. No partial effects were observed for intensities ranging between *ThreshI-*DES and *SupraThreshI-*DES suggesting that speech arrest occurs under the all-or-none principle. If instead of delivering DES prior to speech initiation, *SupraThreshI-*DES was applied on Broca’s area *during* ongoing word pronunciation (2 patients), it failed to induce any effect (on speech and on language) so that the task was correctly performed.

Quantitative analysis of EMG activity of muscles, comparing the DES-OFF and the *SupraThreshI-*DES interference, showed a significant difference between EMG signal recorded in the two conditions: mean and median frequencies, and PS area, as well as mean and peak RMS values, were all significantly different (*p* < 0.05). On the other hand, EMG signal recorded during *SupraThreshI*-DES stimulation was superimposable to EMG recorded at baseline, suggesting that, during stimulation, no attempt at activating the muscles had occurred. Confirming this observation, no significant differences (*p* > 0.05) were observed when comparing all the EMG parameters (RMS and PS) recorded during *SupraThreshI*-DES with those measured at baseline (**Figure 3B** and **Supplementary Table S1B**). This result indicates that *SupraThreshI*-DES on Broca’s area *prevents* the onset of the motor program rather than *disrupting* its execution. Coherently, when *SupraThreshI*-DES was applied during the already ongoing articulation of the word (2 patients), the EMG parameters were not different (*p* > 0.05) from those recorded in DES-OFF, and no effect on performance was observed.

In 2 out of 20 patients, it was possible to directly stimulate fibers running subcortically below Broca’s cortex with *SupraThreshI*-DES (due to time-restraints of the surgical setting it was not possible to also add a *ThreshI-DES* stimulation). The effect of stimulation of these fibers was superimposable to the effect of *SupraThreshI-*DES delivered at cortical level, resulting in all the parameters of the EMG signal being the same as those of the baseline (*p* > 0.05).

In conclusion, both qualitative and quantitative analysis of the intraoperative data demonstrate that LF-DES delivered at threshold intensity (*ThreshI*-DES), while effective on the neighboring vPM and M1, was completely ineffective on Broca’s area. In order to be effective in inducing “speech arrest” in both naming and counting tasks, current intensity had to be increased (*SupraThreshI-*DES), and in any case this was only effective if delivered before, not during, the tasks. This data demonstrates that “speech arrest” derives from a lack of activation of the motor program, not from an arrest of ongoing execution.

### 3D Map of the *SupraThreshI*-DES Positive Sites on Broca’s Area: Neuroimaging Analysis

**FIGURE 4:**
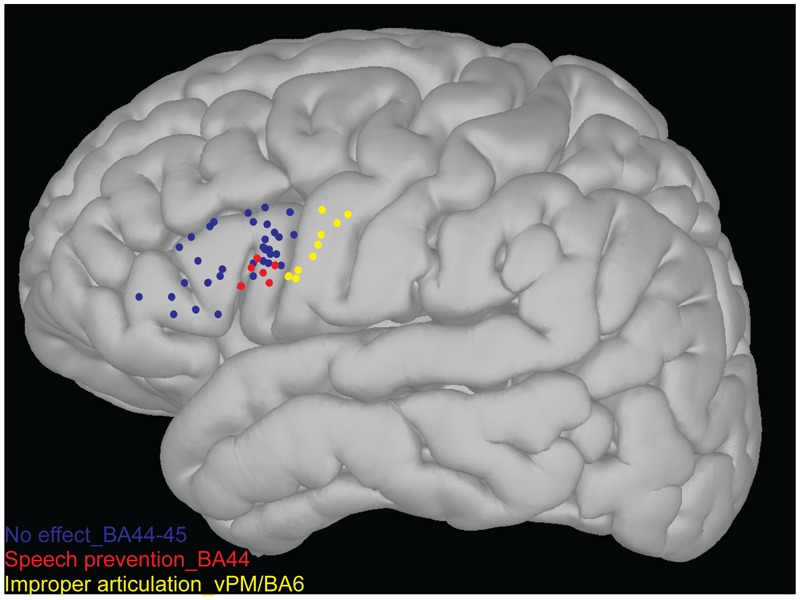
3D map of the stimulation points. Each sites represented over 3D map (non-linear MNI152) as blue, red and yellow points were never responsive to stimulation (both *ThreshI-*DES and *SupraThreshI-*DES) for semantic and phonological errors. Each sites represented onto Broca’s area (BA44-45) as blue points were never responsive to stimulation (both *ThreshI-*DES and *SupraThreshI-*DES) for motor alteration of speech. Each sites represented on more ventral portion of the BA44 as red dots were responsive to *SupraThreshI*-DES (6 patients while 1 patient was excluded due to screen shot inaccuracy), inducing the phenomenon of “speech prevention.” Each sites represented onto caudal cortex of the Broca’s area (the ventral pre-motor cortex, vPM/BA6) as yellow points were responsive to stimulation (*ThreshI*-DES). In these sites, the *ThreshI*-DES induced an improper articulation of the speech.

The topographical distribution of the effect of LF-DES was created on an average 3D map plotting the position of each “eloquent” site, in each patient (see Materials and Methods). Only the sites responsive with a clear effect on task performance to at least three stimulations were considered “eloquent” and therefore reported on the map. All eloquent sites were plotted for comparison on the same map (**Figure 4**). Two main results emerged from this analysis. First, the topographical distribution of sites where *ThreshI*-DES was successful in impairing the task by inducing an improper articulation of speech (yellow dots in **Figure 4**) clustered, as expected, in vPM, with no eloquent sites found on Broca’s area, when stimulated at this intensity (blue dots in **Figure 4**). Second, it emerged that the effect of *SupraThreshI*-DES on task performance, i.e., “speech prevention,” was actually located on Broca’s area, although not homogeneously distributed on BA44/45, but clustering the ventral portion of BA44 sector (vBA44, red dots in **Figure 4**). The quantitative analysis, matched with the topographic distribution of LF-DES eloquent sites, showed that all the eloquent sites on Broca’s area were characterized by the abortion of any attempt to speak, and that the EMG recorded during stimulation was not different from the EMG recorded at baseline. The disruption of an ongoing EMG activity was never observed. Interestingly, the stimulation of all sites reported on the 3D map failed also to elicit semantic and phonological errors; when applied to Broca’s area before speech onset, both *ThreshI-*DES and *SupraThreshI-*DES failed to induce either semantic or phonological errors.

The overall map shows that the effect of LF-DES on Broca’s area on language production is limited to the ventral BA44, that a higher intensity of stimulation is needed compared to other cortical motor areas and that, when effective, stimulation results in “speech prevention” rather than in “speech arrest,” since LF-DES does not arrest ongoing speech, but prevents its onset.

**FIGURE 5:**
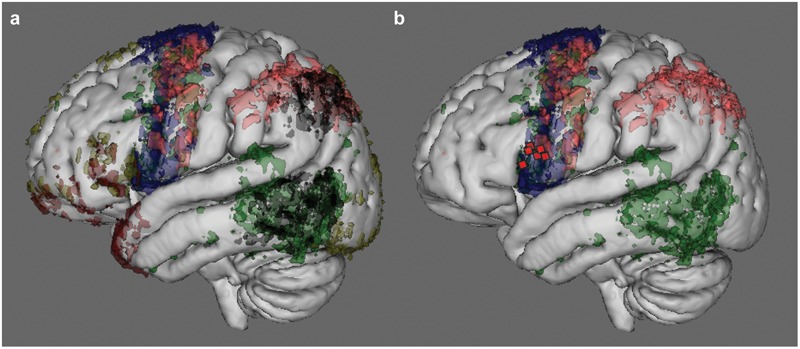
Cortical termination of the main language fibers, as demonstrated by MR tractography. Results of the MR Tractography performed on six out of the seven patients that received *SupraThreshI*-DES. The panel **(a)** shows the cortical terminations, in voxels, that were reached by the main systems of fibers reported as strictly associated to language function (including speech) by the neurosurgical literature. The color code refers to the number of patients in which fiber cortical termination sites were found. The higher the number of patients the higher is the intensity of the color. Voxels representing only one patient were excluded. The main tracts represented were: arcuate fasciculus (AF, in green), the superior longitudinal fasciculus component II and III (SLF II-III, in pink), the superior longitudinal fasciculus component temporo-parietal (SLF-tp, in black), the frontal aslant tract (FAT, in blue), the inferior fronto-occipital fasciculus (IFOF, in yellow) and the uncinate fasciculus (UF, in red). The terminations of AF, of SLF II-III and of FAT are shown in panel **(b)**: in the inferior frontal gyrus, these tracts reach the ventral portion of BA44 where the stimulation sites related to “speech prevention” phenomenon (red squares) were found.

Since only a supra-threshold intensity stimulation of Broca’s area was effective in blocking eloquence, the above results are compatible with the hypothesis that subcortical fibers might have been involved rather than, or in addition to, the cortical site *per se*. In order to understand which of the main systems of fibers involved in language might have been included when stimulating Broca’s area, the topographic distribution of the eloquent sites on vBA44 was matched with the probabilistic map of terminations, within the frontal lobe, of the main systems of fibers sub-serving the language network, as demonstrated by High-Angular Resolution Diffusion Imaging (HARDI) *q*-ball technique (see Materials and Methods). The main white matter bundles of the language network were reconstructed in six patients and the cortical terminations of tractography analysis plotted in the MNI space (**Figure 5a**). This analysis highlights the terminations of the arcuate fasciculus (AF), of the superior longitudinal fasciculus component II and III (SLF II-III) and of the frontal aslant tract (FAT), all reaching the ventral portion of BA44 (**Figure 5b** in green, pink and blue respectively) where the stimulation sites related to the “speech prevention” phenomenon were found (red squares in **Figure 5b**). Parallel to their termination in vBA44, the same systems of fibers emerge also in the ventral portion of vPM, where DES induced the improper articulation of speech.

## Discussion

Motor control of speech requires highly skilled voluntary activation of up to 100 phono-articulatory muscles driven by bulbar motoneurons, controlled bilaterally by the primary motor cortex (M1) (Ackermann and Riecker, 2010). For more than 100 years, Broca’s area has dogmatically been considered to be a peculiar motor cortex in charge of motor control of language production (BA44–45) (Berker et al., 1986), despite the fact that conclusive evidence supporting its motor properties has never been provided. Today, attributing Broca’s area with a direct role in motor control of speech production is highly debated due to a lack of a univocal correlation between “production” aphasia and pure lesions of this area. In this respect, “apraxia of speech” is a paradigmatic example, pointing to vPM/BA6 rather than Broca’s area as the motor area of speech: in fact, it is a clear phono-articulatory dysfunction resulting from heterogeneous lesions, which have the ventral pre-motor cortex (vPM), and not BA44–45, as a common feature (New et al., 2015). Mirroring this observation, and supporting the debate, is the evidence that injuries restricted to Broca’s area do not result in a permanent motor deficit, as is the unfortunate rule regarding damage to motor cortical areas, but rather in a temporary mutism (Mohr et al., 1978). On the other hand, should a putative direct role of Broca’s area in motor control of speech be hypothesized, it could be exerted either by shaping the activity of M1 or as the independent control of bulbar motoneurons. In both cases, Broca’s area must affect motoneuronal excitability and, in turn, the activity of phono-articulatory muscles, either directly or indirectly via M1. In both cases, evidence for a functional connection of Broca’s area with motoneurons driving phono-articulatory muscles must be provided and is at present lacking.

An alternative point of view, possibly adding interesting elements to address this issue, comes from the modern linguistic theory (Hickok and Poeppel, 2004, 2007; Papoutsi et al., 2009; Long et al., 2016). It postulate that, within the language cortical network, the ventral part of BA44 might compute the phonetic encoding (Papoutsi et al., 2009; Long et al., 2016), i.e., the pre-articulation process translating syllables into articulatory gestures, which are then organized by the ventral premotor-primary motor (vPM-M1) areas directly controlling the recruitment of the phono-articulatory muscles. Accordingly, a pure lesion to ventral BA44, would block speech production by preventing the phonetic translation from Broca’s area to vPM-M1. A direct investigation of this model in ecological conditions is extremely difficult, although not completely impossible. Neurosurgical literature reports interesting observations recorded during surgical resection of brain tumors performed with the brain mapping technique (Bello et al., 2006; Tate et al., 2014, 2015; Chang et al., 2017). This technique allows the opportunity to stimulate, with direct electrical current (DES), cortical areas and subcortical fibers. In this setting the frontal operculum, the central and precentral gyri have been extensively investigated in awake patients performing object picture naming and/or counting tasks. When stimulating Broca’s area, neurosurgeons report an impairment of the task, an effect known as “speech arrest” (Luders et al., 1987; Axelson et al., 2009; Chang et al., 2011; Tate et al., 2014) and described as “the block of ongoing speech in absence of oro-facial movements or vocal output” of patients. The definition of the speech arrest reported so far is based on the clinical observation of the behavior of patients during surgery, and it seems coherent with the modern linguistic model (Hickok and Poeppel, 2004, 2007; Papoutsi et al., 2009; Long et al., 2016). Although very interesting, the clinical/behavioral observation is not, *per se*, sufficient to unravel whether the transient lesion induced in Broca’s area by DES results in a block of the pre-articulation phonetic encoding to be transmitted to vPM-M1 or rather in a block of the direct action of Broca’s on motor unit recruitment in phono-articulatory muscles. In absence of EMG recording during the task, it is not possible to distinguish between these two conditions, which are very different in terms of motor control. The phenomenological clinical observation, based solely on auditory and visual inspection of vocal output and motor contraction of facial muscles, cannot provide a causal, mechanistic model of the neural processes leading to impairment of speech. Moreover, in the neurosurgical literature “anarthria” and “speech arrest,” obtained when DES is applied to vPM or to Broca’s area respectively, are reported as synonymous (Chang et al., 2011; Tate et al., 2014; Mandonnet et al., 2016), although very different phenomena. A recent study by our group revealed that the effect of DES on the two areas is actually very different in terms of muscle behavior (Cerri et al., 2015). Visual inspection of EMG activity suggests indeed that DES delivered on vPM before the onset of speech, disrupts the ongoing muscle activity, while when delivered on Broca’s area before the onset of speech it halted speech by preventing the activation of the motor program altogether, not by disturbing its ongoing execution. Although very interesting, this descriptive report was not sufficient to shed definitive light on this issue, because it did not exclude with a specific and quantitative analysis the direct action of Broca’s area on muscle activity. This was the goal of the present study, to investigate, with a quantitative approach, the effect of DES-induced transient inactivation of Broca’s area on EMG activity of phono-articulatory muscles recorded before and during speech production, in object picture naming and counting tasks. Overall the results confirm the previous study (Cerri et al., 2015), challenging a direct role of Broca’s area in modulating the recruitment of phono-articulatory muscles’ motor units. When stimulation was delivered before the onset of speech, indeed no active inhibition or disruption (as sort of dysfunctional recruitment) of the muscle activity was observed, all muscles remained instead relaxed in the resting state (baseline condition), and speech never started. Conversely, if the stimulation occurred during ongoing speech production it was ineffective and objecting naming or counting proceeded normally. This quantitative study is strongly supported by the complementary evidence that Broca’s area does not have a motor output in the resting condition (Cerri et al., 2015) and thus cannot be defined as a proper “motor” area. Consistently, our data raise the doubt on the existence of a functional connection between Broca’s area and the motor nuclei controlling phono-articulatory muscles and suggests that this area may instead be involved in more cognitive pre-articulatory function.

Based on our results, we suggest that the term “speech arrest” be reworded to “speech prevention,” to give a more accurate description of the fact that DES on Broca’s area *prevents* the onset of muscle activation, rather than arresting it. Moreover, according to intraoperative observation, this “speech prevention” induced by the DES is an “*all-or-none”* phenomenon, in that this deficit either occurred fully, or not at all, without partial deficits. We interpret this finding with the hypothesis that Broca’s area may operate as a *functional gate*, authorizing the phonetic translation preceding speech articulation. When applied to Broca’s area, DES may interfere with its computational activity inactivating the *functional gate*, and thus the phonetic encoding would be prevented, eventually halting the naming process. The suggested role of Broca’s area in gating the phonetic encoding is supported by data reported by Flinker et al. (2015), showing that Broca’s activation occurs few milliseconds before the actual motor program execution activating motor cortices, reasonably pointing to a role in the phonetic coding rather than in semantic/phonological coding, which is expected to occur earlier with respect to Broca’s activation.

This hypothesis fits the most accredited current linguistic model of speech production (Hickok and Poeppel, 2004, 2007; Papoutsi et al., 2009; Long et al., 2016). Coherently, DES-induced ‘speech prevention’ sites are clustered in the ventral-posterior sector of Broca’s area, vBA44, proposed in this model as the best candidate for phonetic encoding, feeding the vPM-M1 network (Papoutsi et al., 2009; Long et al., 2016).

According to our data, “speech prevention” is an intensity-dependent effect: the intensity of *ThreshI*-DES, effective in inducing speech disruption when applied to the motor cortices neighboring Broca’s area (vPM and M1) (Cerri et al., 2015), was indeed ineffective on Broca’s area, and a significantly higher intensity (*SupraThreshI*-DES) was required to induce “speech prevention.” The hypothesis that vBA44 is similar in function to the other motor cortices but just less excitable, seems simplistic, too “*ad hoc*” and unjustified, especially given the structural cyto-architectonic similarities between BA44 and vPM (Amunts et al., 1999). Alternatively, “speech prevention” might actually result from inactivation of subcortical fibers running below the cortical site of stimulation. Should this be true, we can speculate that the effect is not elicited with *ThreshI*-DES because the current is not strong enough to reach and act on the subcortical fibers, which instead it can do when *SupraThreshI-*DES is utilized. DES delivered subcortically to vBA44 indeed induced the same effect as *SupraThreshI-*DES delivered cortically. The conceptual consequences of this hypothesis depend on the nature of the axons running subcortical to vBA44. If these fibers are in direct connection with Broca’s area, afferents or efferents, then “speech prevention” can still be attributed, although indirectly, to Broca’s area, though with the important new evidence that prevention does not derive from a motor impairment. Conversely, if the stimulated axons are running on their way to other targets bypassing vBA44, the attribution of “speech prevention” to the effect of DES disrupting the activity of Broca’s cortical neurons, and consequently their hypothesized role in phonetic translation, must be reconsidered. Surprisingly, we found that *SupraThreshI*-DES applied before the onset of speech also failed to induce any effect on language (semantic and phonological processing). The answer may be found in the system of fibers belonging to the language network and reaching the frontal lobe, described with the most advanced neuroimaging techniques (Catani et al., 2013): the arcuate fasciculus (AF), the superior longitudinal fasciculus component II and III (SLF II-III), inferior frontal occipital fasciulus (IFOF), uncinate fasciculus (UF) and the frontal aslant tract (FAT), all reported to be involved in different functions in language network. These range from phonological (Dick and Tremblay, 2012; Chang et al., 2015; Fujii et al., 2015, 2016), syntactic (Chang et al., 2015; Skeide et al., 2016), reading (Gullick and Booth, 2015), repetition (Chang et al., 2015) processes, starting mechanisms of speech (Boetz and Barbeau, 1971; Kinoshita et al., 2015), to phono-articulation (Chang et al., 2015). Building on this knowledge, we used HARDI tractography to reconstruct the white matter connectins of Broca’s area (**Figure 5a**) to disclose whether these may be reached by the DES. Our analysis, consistent with the most recent post-mortem microdissection studies (Lemaire et al., 2013; Sarubbo et al., 2016), shows parallel branches of AF, SLF II-III and FAT (**Figure 5b**), running below the sites of stimulation in BA44 and terminating in both BA44 and in the neighboring vPM, all potentially responsible for the “speech prevention” effect, although current spread might not reach the FAT tract as easily, due to its medial and deep course (Sarubbo et al., 2016). The observation that DES on vPM induces speech motor disruption (Cerri et al., 2015), an effect different from “speech prevention,” suggests that the branches reaching vBA44, rather than those reaching vPM could be involved in the effect. Should this be the case, however, DES would be expected to be equally effective when acting on the AF and SLF-III vBA44 branches (*SupraThreshI-*DES) feeding Broca’s area by interrupting transmission through their axons on their way to Broca’s and/or when acting (*ThreshI-*DES) on Broca’s cortex by interrupting the activity of the cortical neurons receiving AF and/or SLF-II-III vBA44 branches. However, in all our tests *ThreshI-*DES was never effective on Broca’s area. Certainly, the complexity of the language network does not allow an easy allocation of function to all the different structures involved, i.e., cortical areas and tracts, with a single study, but considering our data the univocal attribution of phonetic translation to Broca’s area alone must be further investigated. Data collected in two patients showing that DES failed to impair the task, when applied during ongoing articulation of the words to be pronounced, must be substantiated with a significantly higher number of trials. Dedicated studies are needed aimed at assessing the time course of the effect of DES at different stages of articulation. This would support and better disclose the role of Broca in gating phonetic encoding.

Finally, the lack of phonological and semantic errors when stimulating all the BA44-45 area with both *ThreshI-*DES and *SupraThreshI-*DES in our study, contradicting the neurosurgical literature (Tate et al., 2014), deserves discussion. This apparent contradiction could be explained considering that many studies evaluate Broca’s function on performance of entire sentences (Démonet et al., 2005), suggesting the involvement of Broca’s area in higher order semantic processes (Hagoort, 2005; Vigneau et al., 2006; Price, 2010; Friederici, 2011), rather than single word tasks. As our data focuses on task performance using single words, our results may actually support this view.

## Conclusion

This study is the first to investigate the effect of DES applied on Broca’s area during speech using a quantitative approach applied to the EMG recorded from phono-articulatory muscles. Results strongly challenge the concept of a direct functional connection existing between Broca’s area and motoneurons of phono-articulatory muscles. We show that rather than inducing interruption/disruption of ongoing speech, commonly referred to as “speech arrest,” intraoperative stimulation of ventral BA44 results in a complete lack of onset of muscle activation, which might be better defined as “speech prevention.” “Speech prevention” is an all-or-none intensity-dependent effect requiring a higher intensity to induce “speech arrest” when compared to neighboring motor cortices. Intraoperative data points to Broca’s area acting as a *functional gate* at the pre-articulatory stage, allowing or halting phonetic translation into a motor program to be organized and executed by the other motor areas. Direct control of Broca’s area on the phono-articulatory apparatus seems unlikely due to the absence of a direct effect on motor unit recruitment. The strict correlation between DES-intensity and speech prevention, might suggest an appropriate interpretation is that inactivation of subcortical fibers is occurring, likely the SLF II-III and/or the AF running below vBA44 and reaching both vBA44 and vPM, rather than cortical neurons themselves. In this light, “speech prevention” might be considered the result of a substantial shut down of phonetic encoding, affecting both object picture naming and counting tasks. The possibility that more than one tract running below vBA44 contributes to the effect cannot be ruled out, and the univocal attribution of phonetic translation to Broca’s area must be reconsidered.

## Author Contributions

VF and GC designed the study and wrote the manuscript. VF and CS collected the data and performed the data analysis. VF and LF helped in statistical analysis. MM and VF developed the MatLab script. LB selected the patients, directed, and together with MRi and FP, executed the surgical procedure and the intraoperative brain mapping. VF, LF, MRo, and LB contributed to the 3D reconstruction. AC performed the HARDI q-ball tractography analysis. LB and PB contributed to data interpretation. All authors contributed in writing the manuscript. GC directed the project.

## Conflict of Interest Statement

The authors declare that the research was conducted in the absence of any commercial or financial relationships that could be construed as a potential conflict of interest. The reviewer IS and handling Editor declared their shared affiliation, and the handling Editor states that the process nevertheless met the standards of a fair and objective review.

## Footnotes

1 http://neuroimage.usc.edu/brainstorm

2 http://trackvis.org

## Supplementary Material

FIGURE S1 EMG parameters submitted to statistical analysis in a single subject. All the 5 EMG parameters calculated for comparison are illustrated. For each muscle, the EMG parameters were calculated in frequency and time domain in the 4 different experimental conditions: DES-ON (*SupraThreshI-DES*)/Speech prevention, Baseline, DES-OFF and DES-ON (*ThreshI-DES*)/No effect. **(Left)** DES-ON (*SupraThreshI-DES*)/Speech prevention vs. Baseline. **(Right)** DES-ON (*ThreshI-DES*)/No effect vs. DES-OFF.

TABLE S1A The table reports the exact *p*-values obtained from statistical comparisons between EMG signals related to DES-off condition and DES-on No effect condition for all analyzed data in each patient (20 patients), for all EMG calculated parameters (PS median and mean frequency and area and RMS mean and peak) and for all recorded muscles (contra- and ipsilateral orbicularis oris, mylohyoid and mentalis muscles and contralateral platysma muscle). Empty cells indicate that the EMG signal was not suitable for analysis due to technical problems.

TABLE S1B The table reports the exact *p*-values obtained from statistical comparisons between EMG signals related to Baseline and Speech prevention condition for all analyzed data in each patient (7 patients), for all EMG calculated parameters (PS median and mean frequency and area and RMS mean and peak) and for all recorded muscles (contra- and ipsilateral orbicularis oris, mylohyoid and mentalis muscles and contralateral platysma muscle). Empty cells indicate that the EMG signal was not suitable for analysis due to technical problems.
