# Supplementary figures and images for: Broca’s Area as a Pre-articulatory Phonetic Encoder: Gating the Motor Program
Source: Front Hum Neurosci. 2018 Feb 22;12:64. doi: 10.3389/fnhum.2018.00064 (PMC5826965; doi:10.3389/fnhum.2018.00064)

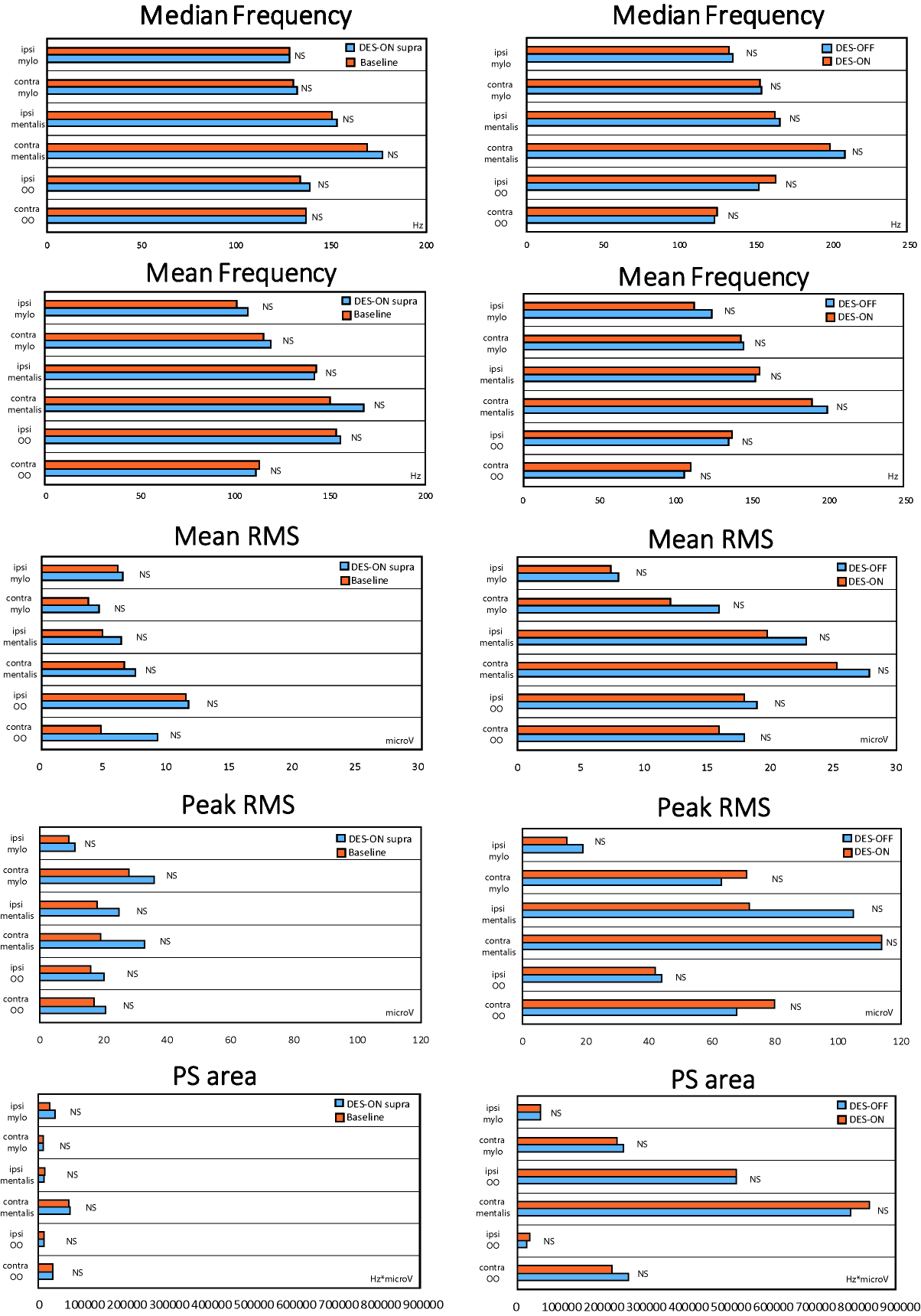

Supplement: FIGURE S1 — EMG parameters submitted to statistical analysis in a single subject. All the 5 EMG parameters calculated for comparison are illustrated. For each muscle, the EMG parameters were calculated in frequency and time domain in the 4 different experimental conditions: DES-ON (SupraThreshI-DES)/Speech prevention, Baseline, DES-OFF and DES-ON (ThreshI-DES)/No effect. (Left) DES-ON (SupraThreshI-DES)/Speech prevention vs. Baseline. (Right) DES-ON (ThreshI-DES)/No effect vs. DES-OFF. [file Image_1.TIFF]
