# Supplementary material for: Broca’s Area as a Pre-articulatory Phonetic Encoder: Gating the Motor Program
Source: Front Hum Neurosci. 2018 Feb 22;12:64. doi: 10.3389/fnhum.2018.00064 (PMC5826965; doi:10.3389/fnhum.2018.00064)
Supplement: TABLE S1A — The table reports the exact p-values obtained from statistical comparisons between EMG signals related to DES-off condition and DES-on No effect condition for all analyzed data in each patient (20 patients), for all EMG calculated parameters (PS median and mean frequency and area and RMS mean and peak) and for all recorded muscles (contra- and ipsilateral orbicularis oris, mylohyoid and mentalis muscles and contralateral platysma muscle). Empty cells indicate that the EMG signal was not suitable for analysis due to technical problems. [file Table_1.PDF]

PS Median Frequency (p value)							
	Ipsi Orbicularis Oris	Contra Orbicularis Oris	Ipsi Mylohyoid	Contra Mylohyoid	Ipsi Mentalis	Contra Mentalis	Contra Platysma
**Patient n° 1**	0,100862	0,266381		0,711026		0,873845	0,427263
**Patient n° 2**	0,179713	0,179713		0,654721			0,550985
**Patient n° 3**	0,880169	0,801614		0,159413	0,615303	0,056189	
**Patient n° 4**	0,138642	0,722283		0,085832			0,858955
**Patient n° 5**		0,257745	0,096939	0,521316	0,939857	0,096939	
**Patient n° 6**	0,456057	0,332564		0,654721	0,456057	0,550985	
**Patient n° 7**	0,509856	0,758416	0,244268	0,860491	0,895133	0,598012	
**Patient n° 8**	0,482406	0,527260	0,140181	0,944000	0,292040	0,622926	
**Patient n° 9**	0,643429	1,000000		0,354540		0,064078	0,643429
**Patient n° 10**	1,000000	0,671126		0,051541	0,174232	0,234483	
**Patient n° 11**	0,521316	0,615303	0,711026	0,533256	0,138642	0,138642	
**Patient n° 12**	0,482406	0,615303	0,244268	0,654721	0,615303	0,234483	
**Patient n° 13**	0,070175	0,368262	0,113096	0,881498	0,050613	0,427263	
**Patient n° 14**	0,660390	0,929976	0,147077	0,895133	0,792069	0,086610	
**Patient n° 15**	0,527260	0,561055		0,361153		0,482406	0,399270
**Patient n° 16**	0,369915	0,212219		0,105698		0,339523	0,537960
**Patient n° 17**	0,357098	0,147857		0,352970		0,065502	0,065502
**Patient n° 18**	0,325404	0,325404	0,067800	0,067800	0,888275	0,574149	
**Patient n° 19**	0,479501	0,479501		0,479501		0,288845	0,479501
**Patient n° 20**	0,196707	0,070702		0,438579		0,438579	
**PS Mean Frequency (p value)**							
	Ipsi Orbicularis Oris	Contra Orbicularis Oris	Ipsi Mylohyoid	Contra Mylohyoid	Ipsi Mentalis	Contra Mentalis	Contra Platysma
**Patient n° 1**	0,124823	0,185790		0,791296		0,596628	0,915700
**Patient n° 2**	0,550985	0,052633		0,881498			0,296718
**Patient n° 3**	0,763025	0,763025		0,056189	0,615303	0,087533	
**Patient n° 4**	0,441269	0,514670		0,173072			0,858955
**Patient n° 5**		0,096939	0,257745	0,650768	0,880056	0,082683	
**Patient n° 6**	0,371094	0,296718		0,654721	0,765595	0,765595	
**Patient n° 7**	0,660390	0,929976	0,147077	0,895133	0,792069	0,086610	
**Patient n° 8**	0,888275	0,888275	0,574149	0,574149	0,122259	0,574149	
**Patient n° 9**	0,354540	1,000000		0,064078		0,064078	0,643429
**Patient n° 10**	0,734095	0,308180		0,126371	1,000000	0,865135	
**Patient n° 11**	0,650768	0,650768	0,791296	0,615303	0,296718	0,296718	
**Patient n° 12**	0,650768	0,888275	0,650768	0,791296	0,765595	0,296718	
**Patient n° 13**	0,354540	0,654721	0,368262	0,456057	0,765595	0,765595	
**Patient n° 14**	0,765595	0,765595	0,514670	0,538473	0,403824	0,660390	
**Patient n° 15**	0,673417	0,160058		0,091825		0,482406	0,122259
**Patient n° 16**	0,532796	0,558709		0,118912		0,755138	0,793832
**Patient n° 17**	0,254214	0,254214		0,655021		0,615303	0,371094

**Patient n° 18**	0,895133	0,792069	0,888275	0,650768	0,888275	0,122259	
**Patient n° 19**	0,313939	0,727964		0,479501		0,313939	0,479501
**Patient n° 20**	0,301700	0,121336		0,698536		0,518605	

PS Area (p value)

	**Ipsi Orbicularis Oris**	**Contra Orbicularis Oris**	**Ipsi Mylohyoid**	**Contra Mylohyoid**	**Ipsi Mentalis**	**Contra Mentalis**	**Contra Platysma**
**Patient n° 1**	0,427263	0,223495		0,491433		0,791296	0,711026
**Patient n° 2**	0,550985	0,654721		0,296718			0,654721
**Patient n° 3**	0,268926	1,000000		0,919944	0,546494	0,268926	
**Patient n° 4**	0,138642	0,313939		0,858955			0,858955
**Patient n° 5**		0,880056	0,365259	0,880056	0,880056	0,762807	
**Patient n° 6**	0,371094	0,654721		0,371094	0,101051	0,293718	
**Patient n° 7**	0,113708	0,312227	0,660390	0,792069	0,538473	0,538473	
**Patient n° 8**	0,325404	0,325404	0,067800	0,067800	0,888275	0,574149	
**Patient n° 9**	0,354540	0,169160		0,354540		0,064078	0,064078
**Patient n° 10**	0,395766	0,865135		0,308180	0,308180	0,089430	
**Patient n° 11**	0,654721	0,491433	0,865135	0,792069	0,296718	0,293718	
**Patient n° 12**	0,550985	0,654721	0,654721	0,880056	0,325404	0,711026	
**Patient n° 13**	0,538473	0,929976	0,880056	0,223495	0,223495	0,678403	
**Patient n° 14**	0,313227	0,433849	0,433849	0,433849	0,263553	0,179713	
**Patient n° 15**	0,091825	0,122259		0,654721		0,673417	0,888275
**Patient n° 16**	0,532796	0,532796		0,296718		0,371094	0,118912
**Patient n° 17**	0,079412	0,079412		0,254214		0,147857	0,254212
**Patient n° 18**	0,509856	0,758416	0,244268	0,860491	0,895133	0,598012	
**Patient n° 19**	0,313939	0,313939		0,313939		0,077101	0,313939
**Patient n° 20**	0,245279	0,245279		0,121336		0,605577	

RMS mean (p value)

	**Ipsi Orbicularis Oris**	**Contra Orbicularis Oris**	**Ipsi Mylohyoid**	**Contra Mylohyoid**	**Ipsi Mentalis**	**Contra Mentalis**	**Contra Platysma**
**Patient n° 1**	0,633839	0,124823		0,368262		0,266381	0,427263
**Patient n° 2**	0,881498	0,233039		0,654721			0,765595
**Patient n° 3**	0,442424	0,546494		0,070441	0,368262	0,368262	
**Patient n° 4**	0,050613	0,07686		0,050613			0,173072
**Patient n° 5**		0,705997	0,227360	0,113096	0,309600	0,070175	
**Patient n° 6**	0,881498	0,881498		0,101051	0,456057	0,765595	
**Patient n° 7**	0,312227	0,064984	0,113708	0,187463	0,058851	0,104017	
**Patient n° 8**	0,070175	0,368262	0,113096	0,881498	0,050613	0,427263	
**Patient n° 9**	1,000000	0,354540		0,925820		0,064078	0,064078
**Patient n° 10**	0,089430	0,234483		0,126371	0,308180	0,057418	
**Patient n° 11**	0,104017	0,064984	0,368262	0,368262	0,881498	0,881498	
**Patient n° 12**	0,354540	0,654721	0,368262	0,456057	0,765595	0,765595	
**Patient n° 13**	0,791296	0,765595	0,546494	0,234483	0,643429	0,244268	
**Patient n° 14**	0,881498	0,881498	0,858955	0,138642	0,881498	0,643429	

**Patient n° 15**	0,482406	0,482406		0,091825		0,399270	0,261055
**Patient n° 16**	0,160491	0,061313		0,174232		0,072931	0,639922
**Patient n° 17**	0,792460	0,792460		0,429907		0,312227	0,309600
**Patient n° 18**	0,881498	0,399270	0,261055	0,929976	0,257745	0,881498	
**Patient n° 19**	0,070441	0,159413		0,174232		0,482406	0,223495
**Patient n° 20**	0,605577	0,796254		0,796254		0,070702	
**RMS peak (p value)**							
	**Ipsi Orbicularis Oris**	**Contra Orbicularis Oris**	**Ipsi Mylohyoid**	**Contra Mylohyoid**	**Ipsi Mentalis**	**Contra Mentalis**	**Contra Platysma**
**Patient n° 1**	0,491433	0,080717		0,560445		0,427263	0,223495
**Patient n° 2**	0,296718	0,881498		0,881498			0,550985
**Patient n° 3**	0,131669	0,919944		0,546494	0,546494	0,687683	
**Patient n° 4**	0,313939	0,138642		0,858955			0,593955
**Patient n° 5**		0,705991	0,290836	0,365259	0,257745	0,939857	
**Patient n° 6**	0,233039	0,881498		0,371094	0,073639	0,179713	
**Patient n° 7**	0,538473	0,403824	0,660390	0,312227	0,929976	0,312227	
**Patient n° 8**	0,206091	0,574149	0,325404	0,160058	0,778729	0,888275	
**Patient n° 9**	0,164916	0,164916		1,000000		0,064078	0,354540
**Patient n° 10**	0,496906	0,126371		0,174232	0,051541	0,061727	
**Patient n° 11**	0,104017	0,223495	0,368262	0,368262	0,881498	0,939857	
**Patient n° 12**	0,881498	0,881498	0,538473	0,929976	0,257745	0,881498	
**Patient n° 13**	0,131669	0,858955	0,312227	0,929976	0,312227	0,312227	
**Patient n° 14**	0,147077	0,895133	0,792069	0,086610	0,546494	0,546494	
**Patient n° 15**	0,399270	0,888275		0,261055		0,091825	0,934150
**Patient n° 16**	0,312227	0,069915		0,660390		0,296718	0,160491
**Patient n° 17**	0,429907	0,560445		0,574149		0,313939	0,496906
**Patient n° 18**	0,257745	0,096939	0,521316	0,939857	0,881498	0,538473	
**Patient n° 19**	0,070441	0,159413		0,131669		0,104017	0,126371
**Patient n° 20**	0,121336	0,403824		0,438579		0,796254	

Tab.1A Supplementary Information: The table reports the exact p-values obtained from statistical comparisons between EMG signals related to DES-off condition and DES-No effect condition for all analysed data in each patient (20 patients), for all EMG calculated parameters (PS median and mean frequency and area and RMS mean and peak) and for all recorded muscles (contra- and ipsilateral orbicularis oris, mylohyoid and mentalis muscles and contralateral platysma muscle). Empty cells indicate that the EMG signal was not suitable for analysis due to technical problems.
