# Supplementary material for: Broca’s Area as a Pre-articulatory Phonetic Encoder: Gating the Motor Program
Source: Front Hum Neurosci. 2018 Feb 22;12:64. doi: 10.3389/fnhum.2018.00064 (PMC5826965; doi:10.3389/fnhum.2018.00064)
Supplement: TABLE S1B — The table reports the exact p-values obtained from statistical comparisons between EMG signals related to Baseline and Speech prevention condition for all analyzed data in each patient (7 patients), for all EMG calculated parameters (PS median and mean frequency and area and RMS mean and peak) and for all recorded muscles (contra- and ipsilateral orbicularis oris, mylohyoid and mentalis muscles and contralateral platysma muscle). Empty cells indicate that the EMG signal was not suitable for analysis due to technical problems. [file Table_2.PDF]

PS Median Frequency (p value)							
	Ipsi Orbicularis Oris	Contra Orbicularis Oris	Ipsi Mylohyoid	Contra Mylohyoid	Ipsi Mentalis	Contra Mentalis	Contra Platysma
**Patient n° 5**		0,429622	0,104965	1,000000	0,803034	0,061391	
**Patient n° 6**	0,125507	0,098961		0,104965	0,479501	0,098961	
**Patient n° 7**	0,660390	0,132007	0,147077	0,538473	0,792069	0,086610	
**Patient n° 11**	0,846022	0,294336	0,472434	0,139974	0,891871	0,969019	
**Patient n° 12**	0,231351	0,801060		0,231351		0,801060	0,614295
**Patient n° 18**	0,803034	0,813664	0,244435	0,939857	0,881498	0,538473	
**Patient n° 19**	0,201244	0,293810		0,132007		0,185616	0,819477
**PS Mean Frequency (p value)**							
	Ipsi Orbicularis Oris	Contra Orbicularis Oris	Ipsi Mylohyoid	Contra Mylohyoid	Ipsi Mentalis	Contra Mentalis	Contra Platysma
**Patient n° 5**		0,244435	0,080818	0,677626	0,405742	0,134524	
**Patient n° 6**	0,157300	0,098961		0,104965	0,813664	0,098961	
**Patient n° 7**	0,170904	0,438579	0,939857	0,792069	0,792069	0,339523	
**Patient n° 11**	0,683412	0,922649	0,741299	0,107000	0,221166	0,496704	
**Patient n° 12**	0,112340	0,525359		0,266381		0,873945	0,427263
**Patient n° 18**	0,244435	0,124823	0,266381	0,368262	0,427263	0,633839	
**Patient n° 19**	0,235334	0,273323		0,120692		0,170904	0,784191
**PS Area (p value)**							
	Ipsi Orbicularis Oris	Contra Orbicularis Oris	Ipsi Mylohyoid	Contra Mylohyoid	Ipsi Mentalis	Contra Mentalis	Contra Platysma
**Patient n° 5**		0,880056	0,067385	0,560575	0,067385	0,762807	
**Patient n° 6**	0,244435	0,244435		0,244435	0,244435	0,244435	
**Patient n° 7**	0,345779	0,113096	0,309600	0,070175	0,750214	0,279069	
**Patient n° 11**	0,891871	0,425914	0,683412	0,712148	0,285486	0,712148	
**Patient n° 12**	0,378782	0,899741		0,801060	0,614295	1,000000	0,3787822
**Patient n° 18**	0,104017	0,080818	0,365259	0,368262	0,881498	0,881498	
**Patient n° 19**	0,137110	0,705991		0,067890		1,000000	0,927265
**RMS mean (p value)**							
	Ipsi Orbicularis Oris	Contra Orbicularis Oris	Ipsi Mylohyoid	Contra Mylohyoid	Ipsi Mentalis	Contra Mentalis	Contra Platysma
**Patient n° 5**		0,705997	0,867935	0,134514	0,212356	0,560575	
**Patient n° 6**	0,212356	0,244435		0,345779	0,238594	0,345779	
**Patient n° 7**	0,898669	0,566616	0,279069	0,610492	0,566616	0,949232	
**Patient n° 11**	0,496704	0,156299	0,573320	0,156299	0,156299	0,573320	
**Patient n° 12**	0,068382	0,528734		0,130571		0,207713	0,207713
**Patient n° 18**	0,399270	0,261055	0,929976	0,368262	0,131669	0,858955	
**Patient n° 19**	0,137110	0,137710		0,135765		0,648077	0,855132
**RMS peak (p value)**							
	Ipsi Orbicularis Oris	Contra Orbicularis Oris	Ipsi Mylohyoid	Contra Mylohyoid	Ipsi Mentalis	Contra Mentalis	Contra Platysma
**Patient n° 5**		0,705991	0,080818	0,365259	0,067385	0,939857	
**Patient n° 6**	0,098961	0,365259		0,098961	0,059347	0,705991	
**Patient n° 7**	0,566616	0,161283	0,444834	0,524311	0,372715	0,308326	
**Patient n° 11**	0,953542	0,573320	0,547169	0,091123	0,953542	0,368262	
**Patient n° 12**	0,377822	0,313501		0,256840		0,528734	0,377822
**Patient n° 18**	0,881498	0,223495	0,368262	0,858955	0,257745	0,881498	
**Patient n° 19**	0,223495	0,223495		0,117623		0,927265	1,000000

Tab.1B Supplementary Information: The table reports the exact p-values obtained from statistical comparisons between EMG signals related to Baseline and Speech prevention condition for all analysed data in each patient (7 patients), for all EMG calculated parameters (PS median and mean frequency and area and RMS mean and peak) and for all recorded muscles (contra- and ipsilateral orbicularis oris, mylohyoid and mentalis muscles and contralateral platysma muscle). Empty cells indicate that the EMG signal was not suitable for analysis due to technical problems.
